# Online TCP Throughput Map Maintenance Under Budget-Constrained Vehicular Sensing

**DOI:** 10.3390/s26175364

**Published:** 2026-08-25

**Authors:** Weiwei Hu, Yuichi Ohsita, Hideyuki Shimonishi

**Affiliations:** 1 Graduate School of Information Science and Technology, The University of Osaka, Toyonaka 560-0043, Osaka, Japan; 2 D3 Center, The University of Osaka, Toyonaka 560-0043, Osaka, Japan; y-ohsita@ist.osaka-u.ac.jp (Y.O.); shimonishi.cmc@osaka-u.ac.jp (H.S.)

**Keywords:** TCP throughput map, online map maintenance, vehicular sensing, sensing-budget constraint, digital twin

## Abstract

A Transmission Control Protocol (TCP) throughput map represents communication quality over road networks and supports communication-aware applications in intelligent transportation systems. Maintaining such a map online is challenging because vehicular measurements are sparse and unevenly distributed, network conditions vary rapidly, and sensing-budget constraints limit the number of vehicles from which onboard communication measurements can be uploaded at each time step. This work addresses online TCP throughput map maintenance under sparse vehicular observations and sensing-budget constraints. To support budget-constrained sensing, we combine discoverability-guided vehicle selection and probabilistic map updating within a digital twin (DT)-assisted vehicular sensing architecture. The resulting sensing-and-mapping method, referred to as Discoverability-aware and Statistical Mapping (DISMAP), maintains a spatio-temporal discoverability map to characterize historical sensing coverage and select vehicles that improve the coverage of under-represented regions. It then uses Gaussian Process Regression (GPR) as a probabilistic mapping engine to estimate the mean TCP throughput and predictive standard deviation, where the standard deviation is adjusted using local vehicle density. Simulation results show that DISMAP reduces the mean absolute error (MAE) and mean standard deviation (MSTD) by up to 23.7% and 37.5%, respectively, and achieves a prediction-interval miss rate (PIMR) of 0.048, which is close to the nominal value of 0.05. These results indicate a favorable balance among prediction accuracy, interval sharpness, calibration, and spatial representativeness across different traffic-density conditions.

## 1. Introduction

With the development of 6G and Intelligent Transportation Systems (ITSs), vehicular networks are expected to support a wide range of communication-intensive and latency-sensitive services, such as cooperative perception, edge-assisted driving, and real-time sensing data exchange [1]. For these applications, Transmission Control Protocol (TCP) throughput is an important indicator of communication quality, as it reflects the end-to-end network condition experienced by upper-layer services and affects the reliability of continuous data delivery [2,3]. In dynamic Internet of Vehicles (IoV) environments, understanding how TCP throughput over road networks evolves in space and time is essential to enable communication-aware functions such as routing, handoff, and task allocation [4]. To support such capabilities, it is necessary to maintain a real-time and predictive representation of TCP throughput over road networks, referred to as a TCP throughput map [5]. Such a map enables vehicles and infrastructure to anticipate communication quality and make communication-aware decisions in advance.

An effective TCP throughput map needs to satisfy several key requirements: (i) wide spatial coverage to capture heterogeneous network conditions across diverse road regions and ensure that critical areas are not overlooked; (ii) timely updates to reflect the fast-changing temporal dynamics caused by vehicle mobility and wireless channel variability; and (iii) uncertainty information to indicate the reliability of predicted throughput under sparse measurements and fast dynamics, enabling confidence-aware decision-making in dynamic environments. Although research directly targeting TCP throughput map maintenance remains limited, related studies have explored communication-performance mapping and reliability-aware prediction in vehicular networks [6,7,8,9]. Graph-based learning approaches can infer network performance over road graphs from vehicle measurements, thereby supporting communication-performance mapping over road networks [6]. However, these graph-based map reconstruction approaches are often designed for offline point-estimate prediction and do not explicitly provide reliability information, which limits their applicability to timely and confidence-aware map maintenance in dynamic vehicular environments. GPR-based wireless delay prediction has also been studied to provide probabilistic outputs in online settings [7]. However, existing GPR-based delay prediction focuses mainly on localized delay prediction and control rather than resource-constrained sensing for maintenance of spatially representative TCP throughput maps from sparse vehicular measurements. Therefore, existing studies do not jointly address online TCP throughput map maintenance, spatially representative sensing, and prediction reliability under sparse vehicular measurements and limited sensing budgets.

Recent advances in Digital Twin (DT) technologies provide a useful system-level context for organizing dynamically updated information in transportation and communication networks [10,11,12]. In vehicular networks, a DT-assisted architecture can maintain an online representation of vehicle mobility, measurement availability, and communication performance from continuously collected vehicular observations. Within this architecture, vehicles equipped with onboard communication modules provide distributed throughput-related measurements, while the infrastructure updates the TCP throughput map based on the uploaded observations. Therefore, the key challenge is how to allocate limited measurement-uploading opportunities and perform uncertainty-aware map maintenance when vehicular measurements are sparse, unevenly distributed, and rapidly changing. This motivates a TCP throughput map maintenance framework that integrates adaptive vehicle selection, spatio-temporal coverage tracking, and probabilistic map updating under limited sensing budgets [13,14,15,16].

In our prior work, we studied DT-assisted TCP throughput map construction and introduced a spatio-temporal vehicle selection mechanism to support adaptive measurement acquisition under partial observability [17]. Although this approach reduces map uncertainty, its selection strategy mainly prioritizes uncertainty reduction and may not sufficiently account for the historical sensing coverage of road regions. As a result, some regions may remain under-represented when only a limited number of vehicles can be selected for measurement uploading.

Building on this prior setting, this paper focuses on online TCP throughput map maintenance under sparse vehicular observations, with the goal of improving spatial representativeness while retaining prediction reliability. To support this objective, we develop Discoverability-aware and Statistical Mapping (DISMAP), a sensing-and-mapping method composed of two coordinated components: spatio-temporal discoverability-based vehicle selection and statistical map updating with calibrated uncertainty. The discoverability component records the historical sensing coverage of road regions and selects vehicles that improve the coverage of under-represented areas without requiring ground-truth throughput values at unobserved locations. The statistical mapping component uses Gaussian Process Regression (GPR) as a practical probabilistic mapping engine to estimate the mean TCP throughput and predictive standard deviation and adjusts the standard deviation using local vehicle density as an observable contextual factor. By combining coverage-aware vehicle selection with calibrated statistical map updating, DISMAP supports online TCP throughput map maintenance with prediction reliability information under limited sensing budgets.

The contributions of this work are summarized as follows:

- We formulate the problem of maintaining a TCP throughput map from sparse and dynamically updated vehicular observations under limited sensing budgets. The formulation emphasizes spatial representativeness, timely map updating, and prediction reliability for communication-aware ITS applications.
- We introduce a DT-assisted sensing-and-mapping framework that allocates limited measurement-uploading opportunities to vehicles contributing to under-represented road regions. The framework uses spatio-temporal discoverability to track historical sensing coverage without requiring ground-truth throughput values at unobserved locations.
- We update the TCP throughput map using Gaussian Process Regression (GPR) as a practical probabilistic mapping engine and adjust the predictive standard deviation using local vehicle density as an observable contextual factor. This design provides both throughput estimates and reliability information under sparse observations.
- We evaluate the proposed DISMAP method under dynamic vehicular scenarios with different traffic densities and sensing budgets. The results show that DISMAP provides a favorable balance among prediction accuracy, interval sharpness, calibration, and spatial representativeness across different traffic-density conditions and sensing budgets.

## 2. Related Work

We review related work from three perspectives: DT-assisted vehicular sensing and network modeling, communication-constrained and sparse-data transportation systems, and communication-performance mapping with prediction reliability.

### 2.1. DT-Assisted Vehicular Sensing and Network Modeling

Recent studies have investigated Digital Twin (DT) techniques for the modeling and management of vehicular communication environments. Li et al. [18] presented a DT-oriented online channel-modeling framework that supports continuous sensing and prediction of time-varying wireless channels, facilitating digital representations of vehicular communication environments. These studies demonstrate the potential of DT-assisted architectures for the maintenance of updated representations of vehicular communication conditions from continuously collected data.

DTs have also been adopted for communication-aware optimization and decision-making in Internet of Vehicles (IoV) systems. Gong et al. [19] designed a DT-enhanced resource allocation framework for integrated sensing and communication in DT-enabled IoV. Ding et al. [20] developed a DT-assisted vehicular framework for joint vehicle connection and beamforming optimization by leveraging DT-side knowledge of vehicular dynamics. Zheng et al. [21] proposed a DT-empowered heterogeneous network selection method with knowledge transfer for vehicular networks. These works show the usefulness of DT-side information for network control, resource allocation, beamforming, and network selection.

However, most existing DT-assisted studies mainly focus on channel modeling, resource allocation, beamforming, or network selection. The problem of maintaining a communication-performance map from sparse and resource-constrained vehicular measurements has received less attention. In particular, online TCP throughput map maintenance requires not only updated communication-performance prediction but also spatially representative sensing and prediction reliability under limited sensing budgets. This motivates a DT-assisted vehicular sensing architecture in which vehicles upload distributed throughput-related measurements and the infrastructure maintains an online TCP throughput map from sparse observations.

### 2.2. Communication-Constrained and Sparse-Data Transportation Systems

Recent studies have highlighted the importance of accounting for imperfect communication conditions in connected-vehicle and traffic-control systems. Zhao et al. [22] investigated adaptive strategy controls for connected and automated vehicle platoons under unstable communication conditions, considering dynamic communication topologies and abnormal data transmission. Wu et al. [23] developed a cross-scale traffic–communication control framework that establishes a feedback mechanism between the traffic system and the communication network by combining communication-aware routing with proactive congestion mitigation. These studies demonstrate that unstable communication and traffic–communication coupling can substantially affect vehicle-control and traffic-management performance. However, they primarily focus on optimizing traffic or vehicle-control decisions rather than constructing and maintaining a spatial communication-performance map from budget-constrained vehicular measurements.

Sparse and low-penetration observations have also been investigated in transportation estimation and prediction. An et al. [24] proposed a Bayesian method for estimating the distribution of queue service time from low-penetration connected-vehicle data and used the estimated distribution for robust traffic-signal retiming. He et al. [25] developed a pedestrian-dynamics-informed deep learning approach for prediction of transportation-hub passenger distributions under sparse observation conditions. These studies demonstrate the value of probabilistic estimation and prior-informed spatiotemporal learning when available observations are limited. Nevertheless, their targets are traffic signal optimization and passenger distribution prediction rather than online communication-performance mapping. In contrast, this work jointly considers budget-constrained vehicle selection, online TCP throughput map maintenance, and uncertainty-aware prediction from sparse vehicular measurements.

### 2.3. Communication-Performance Mapping and Prediction Reliability

Communication-performance mapping has been studied as a way to represent network conditions over road networks. Graph-based learning approaches have been used to construct vehicular latency maps by inferring network performance over road graphs from vehicle measurements [6]. Such methods can support spatial coverage across road networks, but they are often optimized for point estimates under offline settings, which limits their ability to adapt to rapid network changes in a timely manner. Moreover, prediction reliability is not always explicitly considered, making it difficult to assess the reliability of the reconstructed map.

Another related direction is probabilistic communication-performance prediction. For example, an uncertainty-aware wireless delay map was constructed by embedding Gaussian Process Regression (GPR) into a model predictive control loop, enabling online delay prediction with confidence-aware outputs [7]. However, this approach mainly focuses on localized delay prediction and control and does not explicitly consider resource-constrained sensing for maintenance of spatially representative communication-performance maps from sparse vehicular measurements. Therefore, existing communication-performance mapping studies are still insufficient for online maintenance of TCP throughput maps when measurements are sparse, spatially biased, and constrained by limited sensing budgets.

In recent years, deep learning-based methods have also been widely used for performance prediction in wireless and sensing systems [26,27,28]. Although these methods can capture complex nonlinear relationships, purely neural models may be less practical in latency-sensitive and highly dynamic IoV scenarios because they often rely on large-scale offline training and may require retraining or continual adaptation when the environmental distribution changes. These limitations motivate lightweight, data-efficient, and reliability-aware modeling methods that can be updated online from limited measurements.

GPR is useful for online map maintenance because it provides both predictions and predictive standard deviations in a data-efficient and interpretable manner. Existing GPR-based methods have been applied to wireless delay prediction and other prediction or optimization tasks [7,29,30]. Several studies have further explored input-dependent uncertainty modeling in GPR to capture heterogeneous noise and varying uncertainty [31,32,33,34]. However, directly modeling input-dependent uncertainty in highly dynamic IoV environments can introduce additional training complexity, especially when measurements are sparse, unevenly distributed, and collected in a rolling manner. Therefore, rather than introducing a complex input-dependent noise model, this work uses GPR as a practical probabilistic mapping engine and applies a lightweight density-aware adjustment to improve prediction reliability for online TCP throughput map maintenance from sparse vehicular measurements.

## 3. System Model

**Figure 1:**
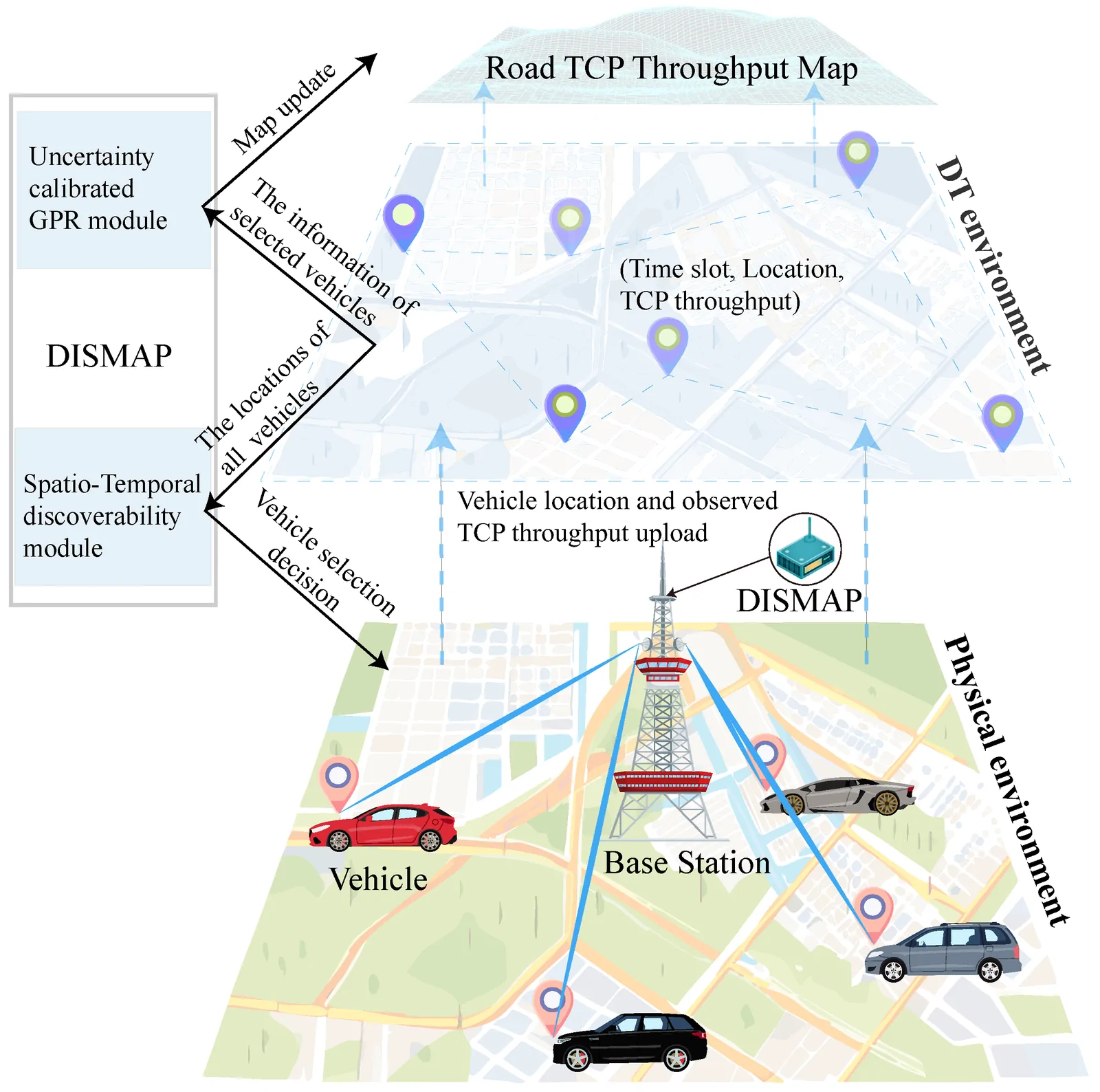
Overview of DT-assisted online TCP throughput map maintenance.

Figure 1 shows the overview of DT-assisted online TCP throughput map maintenance. The system consists of a physical environment and a DT environment deployed at the base station. The DT environment maintains key digital representations of the physical IoV system (i.e., the vehicular network and its road-network communication environment), enabling dynamic sensing decisions and real-time TCP throughput map construction. In the physical environment, a set of vehicles ($V=\{v_{1},\ldots ,v_{M}\}$) that move within a road area are capable of sensing their own position, as well as the local TCP throughput. Several pre-defined observation points ($G=\{g_{1},\ldots ,g_{N}\}$) are uniformly distributed along road networks and serve as representative locations to estimate the spatio-temporal dynamics of TCP throughput. Since the road network is inherently an irregular graph rather than a regular two-dimensional plane, we place observation points along the roads as representative anchors for map construction instead of adopting dense grid discretization. This design avoids excessive spatial discretization while enabling tractable learning and real-time updates under limited sensing resources. Given the spatial scale of the deployment, we treat the aggregated measurements at all observation points as a surrogate for the continuous TCP throughput map over the entire area.

At each time step (t∈T), the base station acquires the positions of all available vehicles and transmits them to the DT environment. In the DT environment, our framework maintains two DTs: the Vehicle DT, which records the spatio-temporal trajectories of all vehicles and the TCP throughput values of the selected vehicles, and the TCP Throughput Map, which constructs the TCP throughput map from the measurements reported by selected vehicles.

The physical and DT environments are orchestrated by DISMAP, which integrates a spatio-temporal discoverability module and a calibrated GPR module. Upon receiving a vehicle position snapshot at time step *t*, DISMAP initiates a closed sensing update loop. First, the spatio-temporal discoverability module assigns each vehicle a discoverability score reflecting its expected contribution to reduced prediction uncertainty and selects, under a given sensing budget, the top-scoring subset, which is then instructed by the base station to upload instantaneous TCP throughput measurements. The collected measurements are ingested by the calibrated GPR module, where a base GPR predictor updates the mean TCP throughput map, while local vehicle-density information is used to calibrate the associated predictive uncertainty. Here, vehicle density serves as an easily observable contextual indicator of local traffic concentration and communication contention. Repetition of this loop at each time step ensures that the system continuously constructs an adaptive and uncertainty-aware TCP throughput map for highly dynamic IoV scenarios.

## 4. DISMAP

### 4.1. Overview

**Notation.** We denote the *n*-th fixed observation point as $g_{n}$ and its (time-invariant) coordinate as $z_{n}$. For vehicles, $v_{m}^{t}$ denotes vehicle *m* at time step *t*, and $z_{m}^{t}$ denotes its coordinate at time step *t*.

In the spatio-temporal discoverability module, each fixed observation point ($g_{n}\in G$) maintains a discoverability score of $D^{t}\left(g_{n}\right)\in \left[0,1\right]$ at time step *t*, which quantifies the remaining sensing demand at that location. The score increases when $g_{n}$ has not been effectively covered by recent throughput reports and decreases when selected vehicles provide informative measurements in its spatio-temporal neighborhood. This evolution is governed by a spatio-temporal decay kernel that weights vehicle observations by spatial proximity and temporal recency, while a small recovery term (δ) prevents long-neglected points from being permanently ignored. Given ${\left\{D^{t-1}\left(g_{n}\right)\right\}}_{g_{n}\in G}$, the module exhaustively evaluates candidate subsets ($S^{t}\subseteq V^{t}$) under $|S^{t}|\leq K^{t}$ and selects the subset that minimizes the updated total discoverability ($\sum_{g_{n}\in G}D^{t}\left(g_{n}\right)$), thereby prioritizing sensing in under-covered regions.

In the prediction module, the TCP throughput reports uploaded by the selected vehicles over the most recent *H* time steps are aggregated into a sliding training buffer. A GP predictor trained on this buffer produces the predictive mean ($\mu^{t}\left(g_{n}\right)$) and the baseline predictive standard deviation ($\sigma_{\mathrm{base}}^{t}\left(g_{n}\right)$) at each observation point. To capture input-dependent uncertainty variations across the road network, we further apply a scaling factor ($\xi_{n}^{t}$) to modulate the baseline uncertainty, yielding $\sigma_{\mathrm{scaled}}^{t}\left(g_{n}\right)=\sigma_{\mathrm{base}}^{t}\left(g_{n}\right)\cdot \xi_{n}^{t}$. This sensing–prediction loop runs in a rolling manner: each selection round updates the discoverability state, which, in turn, guides subsequent vehicle selection, forming a closed feedback loop that progressively improves coverage and map quality in dynamic vehicular environments.

Algorithm 1 summarizes the training process of DISMAP. The detailed formulations are presented in the subsequent subsections.
**Algorithm 1:** DISMAP
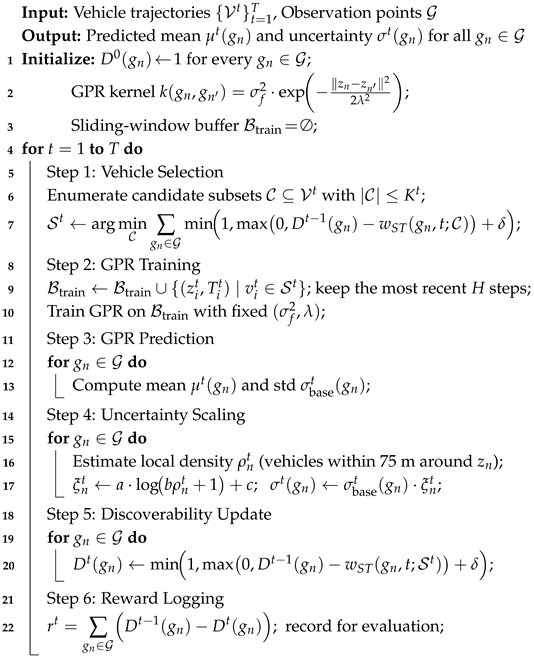


### 4.2. Spatio-Temporal Discoverability Module

Discoverability Score

To steer vehicle selection toward under-observed or long-neglected regions, we introduce a dynamic and continuous measure called the discoverability score. It quantifies the remaining sensing demand of each fixed observation point ($g_{n}$) based on recent spatio-temporal interactions with nearby vehicle reports. Unlike binary coverage indicators, the discoverability score provides a smooth and decaying representation of information deficiency, enabling more informed and adaptive decision-making.

Each observation point ($g_{n}$) maintains a discoverability score, which evolves over time to reflect how well the location has been covered. At each time step (*t*), the score is updated by considering (i) the aggregated spatio-temporal influence from recent vehicle observations and (ii) a gradual recovery term (δ). Specifically, informative coverage decreases the score, while δ allows long-neglected points to regain priority over time. The unified update rule is

(1) $$D^{t}\left(g_{n}\right)=\min\left\{1,\;\max\left(0,\;D^{t-1}\left(g_{n}\right)-w_{ST}\left(g_{n},t;S^{t}\right)\right)+\delta \right\},$$

where the history-aware aggregated influence is computed over a sliding window of the most recent *H* time steps as

(2) $$w_{ST}\left(g_{n},t;S^{t}\right)=\sum_{\tau =t-H}^{t}\;\sum_{v_{m}^{\tau }\in S^{\tau }}w_{ST}\left(v_{m}^{\tau },g_{n};t\right).$$

The spatio-temporal influence of a throughput observation reported by vehicle $v_{m}^{\tau }$ at time τ (with coordinate $z_{m}^{\tau }$) on an observation point ($g_{n}$, with a fixed coordinate of $z_{n}$), evaluated at the current time step (*t*), is defined by

(3) $$w_{ST}\left(v_{m}^{\tau },g_{n};t\right)=\beta^{2}\exp\;\left(-\frac{∥z_{m}^{\tau }-z_{n}{∥}^{2}}{2l_{s}^{2}}\right)\exp\;\left(-\frac{t-\tau }{2l_{t}}\right),$$

where β is a scaling coefficient and $l_{s}$ and $l_{t}$ control the spatial and temporal decay rates, respectively. This formulation assigns greater influence to closer and more recent observations, thereby reducing the discoverability of well-covered locations.

Vehicle Selection Strategy

At each time step (*t*), the goal is to select a subset of vehicles ($S^{t}\subseteq V^{t}$) that minimizes the total discoverability over all observation points. This can be formulated as the following constrained optimization problem:

(4) $$\begin{array}{cc} \min_{S^{t}}\; & \sum_{g_{n}\in G}D^{t}\left(g_{n}\right)\\ s.t.\; & |S^{t}|\;\leq K^{t},\\ \; & S^{t}\subseteq V^{t}, \end{array}$$

where $K^{t}$ is the sensing budget (maximum number of selected vehicles) and $V^{t}$ is the set of vehicles available at time step *t*. By minimizing the cumulative discoverability, the algorithm encourages sensing in regions with the highest information demand.

Sequential Decision-Making View

The vehicle selection process can be viewed as a sequential decision-making problem, where each time step corresponds to the choice of an informative subset of vehicles based on the current coverage status. Concretely, we define the following:

- State $s^{t}$: The current discoverability values of all observation points and the coordinates of all available vehicles, i.e.,
  (5) $$s^{t}=\left({\left\{D^{t-1}\left(g_{n}\right)\right\}}_{n=1}^{N},\;{\left\{z_{m}^{t}\right\}}_{m=1}^{M^{t}}\right),$$
  where $M^{t}$ is the number of available vehicles at time step *t*;
- Action $a^{t}$: The selected vehicle subset for sensing, i.e.,
  (6) $$a^{t}=S^{t}\subseteq {\left\{v_{m}^{t}\right\}}_{m=1}^{M^{t}};$$
- Reward $r^{t}$: The total reduction in discoverability after applying the update in (1), i.e.,
  (7) $$r^{t}=\sum_{n=1}^{N}\left(D^{t-1}\left(g_{n}\right)-D^{t}\left(g_{n}\right)\right).$$

### 4.3. GPR with Density-Aware Uncertainty Calibration

After selecting the optimal vehicle subset ($S^{t}$) at time step *t*, DISMAP uses the TCP throughput measurements uploaded by these vehicles to construct a predictive throughput map over the road network. To obtain both point estimates and confidence under sparse and uneven vehicular sampling, we model the throughput field using GPR [35]. Specifically, GPR yields a posterior predictive distribution at each fixed observation point ($g_{n}$, with a coordinate of $z_{n}$), from which we derive the predicted mean ($\mu^{t}\left(g_{n}\right)$) and a baseline uncertainty estimate ($\sigma_{\mathrm{base}}^{t}\left(g_{n}\right)$), supporting confidence-aware map construction and subsequent uncertainty-informed updates.

We place a Gaussian process prior over the latent throughput field:

(8) $$f\left(\cdot \right)∼\mathrm{GP}\;\left(0,\;k(\cdot ,\cdot )\right),$$

where the mean function is assumed to be 0 and the covariance function is defined using a radial basis function (RBF) kernel:

(9) $$k\left(g_{n},g_{n^{\prime }}\right)=\sigma_{f}^{2}\cdot \exp\;\left(-\frac{∥z_{n}-z_{n^{\prime }}{∥}^{2}}{2\lambda^{2}}\right),$$

where $\sigma_{f}^{2}$ is the signal variance and λ is the spatial length scale. Both hyperparameters are fixed to avoid overfitting in highly dynamic vehicular environments.

To increase stability and smoothness, the training buffer aggregates the measurements from the most recent *H* time steps:

(10) $$B_{\mathrm{train}}^{t}=\left\{\left(z_{i}^{\tau },T_{i}^{\tau }\right)\;|\;\tau =t-H,\ldots ,t,\;v_{i}^{\tau }\in S^{\tau }\right\},$$

where $z_{i}^{\tau }$ and $T_{i}^{\tau }$ denote the coordinate and observed TCP throughput of vehicle *i* at time step τ, respectively.

Let $X_{\mathrm{train}}^{t}$ and $T_{\mathrm{train}}^{t}$ denote the training inputs and outputs extracted from $B_{\mathrm{train}}^{t}$, respectively. The predictive distribution of the TCP throughput at the fixed observation point ($g_{n}$) at time step *t* is Gaussian:

(11) $$T^{t}\left(g_{n}\right)∼N\;\left(\mu^{t}\left(g_{n}\right),\;{\left(\sigma_{\mathrm{base}}^{t}\left(g_{n}\right)\right)}^{2}\right).$$

We define

(12) $$A=K\left(X_{\mathrm{train}}^{t},X_{\mathrm{train}}^{t}\right)+\sigma_{\mathrm{noise}}^{2}I,$$

where $\sigma_{\mathrm{noise}}^{2}$ is the observation noise variance in the base GPR. Therefore, the predictive mean and baseline variance admit the following closed-form expressions:

(13) $$\begin{array}{cc} \mu^{t}\left(g_{n}\right) & =K\left(g_{n},X_{\mathrm{train}}^{t}\right)\;A^{-1}\;T_{\mathrm{train}}^{t}, \end{array}$$

(14) $$\begin{array}{cc} {\left(\sigma_{\mathrm{base}}^{t}\left(g_{n}\right)\right)}^{2} & =k\left(g_{n},g_{n}\right)-K\left(g_{n},X_{\mathrm{train}}^{t}\right)\;A^{-1}\;K\left(X_{\mathrm{train}}^{t},g_{n}\right). \end{array}$$

Here, k(·,·) denotes a positive-definite kernel function. $K(g_{n},X_{\mathrm{train}}^{t})$ is the kernel vector with entries pf $k(g_{n},x_{i})$, and $K(X_{\mathrm{train}}^{t},X_{\mathrm{train}}^{t})$ is the kernel matrix with entries of $k(x_{i},x_{j})$.

Although the base GPR uncertainty reflects the spatial distribution of training samples, it does not fully account for local communication dynamics that affect TCP throughput variability. In vehicular networks, regions with different traffic densities may exhibit different levels of channel contention, cell load, and throughput fluctuation. Therefore, directly using $\sigma_{\mathrm{base}}^{t}\left(g_{n}\right)$ may lead to uncertainty mismatch across different traffic conditions.

To improve the reliability of the predictive uncertainty while preserving the stability of the base GPR predictor, we introduce a density-aware uncertainty calibration mechanism. The local vehicle density ($\rho_{n}^{t}$) is used as an easily observable contextual indicator of local communication dynamics. Instead of directly treating density as uncertainty, we map it to a scaling factor:

(15) $$\xi_{n}^{t}=a\cdot \log\left(b\cdot \rho_{n}^{t}+1\right)+c,$$

where (a,b,c) are calibration parameters learned from ground-truth throughput samples collected during an initial calibration period. Since $\rho_{n}^{t}\geq 0$ and a,b,c>0, we have $\log(b\rho_{n}^{t}+1)\geq 0$ and, hence, $\xi_{n}^{t}=a\log\left(b\rho_{n}^{t}+1\right)+c\geq c>0$. Therefore, the scaling factor and the calibrated predictive standard deviation are always strictly positive.

The logarithmic form is adopted as a compact parametric calibration function for the mapping of local vehicle density to uncertainty scaling.

The (a,b,c) parameters are calibrated using the ground-truth throughput samples of all 200 fixed observation points collected during the initial 25-time-step calibration period to match a target prediction-interval coverage probability (PICP) [36]. After calibration, these parameters are fixed throughout the subsequent evaluation period. This makes the scaling process a quantitative uncertainty calibration procedure rather than an ad hoc adjustment. This design allows the scaling behavior to be adjusted according to empirical calibration samples while maintaining a smooth and monotonic relationship between density and uncertainty.

The final calibrated predictive distribution is defined as follows:

(16) $$T^{t}\left(g_{n}\right)∼N\;\left(\mu^{t}\left(g_{n}\right),\;{\left(\sigma_{\mathrm{cal}}^{t}\left(g_{n}\right)\right)}^{2}\right),$$

where

(17) $$\sigma_{\mathrm{cal}}^{t}\left(g_{n}\right)=\sigma_{\mathrm{base}}^{t}\left(g_{n}\right)\cdot \xi_{n}^{t}.$$

The (a,b,c) parameters are calibrated using the ground-truth throughput samples from the initial calibration period to match a target PICP. This makes the scaling process a quantitative uncertainty calibration procedure rather than an ad hoc adjustment.

Overall, the proposed density-aware uncertainty calibration preserves the original GPR-based mean prediction while improving the reliability of uncertainty estimation under different traffic conditions. The calibrated uncertainty is then used for confidence-aware throughput map construction and fed back to the discoverability-guided vehicle selection module, forming a closed sensing–prediction loop in DISMAP.

## 5. Simulation Evaluation

### 5.1. Simulation  Settings

To enable a controlled and reproducible evaluation with time-synchronized spatio-temporal ground truth, we adopt an OMNeT++ 5.6.2, SUMO 1.8.0, and Veins 5.2 co-simulation environment. SUMO generates vehicle trajectories over the road network, which are synchronized with OMNeT++ through the TraCI interface provided by Veins [37]. The simulated area corresponds to the Toyonaka campus of the University of Osaka, bounded by latitude [34.800667, 34.806915] and longitude [135.446203, 135.457865].

A Shannon-capacity-based throughput proxy is dynamically generated at each time step. For an observation point at distance *d* from the base station, the distance-dependent path loss is calculated as

(18) $$L\left(d\right)=10\eta \log_{10}\left(d+1\right)+L_{\mathrm{abs}}+L_{\mathrm{sca}},$$

where *d* denotes the numerical value of the distance measured in meters; η is the path-loss exponent; and $L_{\mathrm{abs}}$ and $L_{\mathrm{sca}}$ are the absorption and scattering losses in dB, respectively. The received power and the dimensionless signal-to-noise ratio are calculated as

(19) $$P_{r}=P_{\mathrm{tx}}-L\left(d\right),$$

and

(20) $$\gamma =10^{(P_{r}-P_{\mathrm{noise}})/10},$$

where $P_{\mathrm{tx}}$, $P_{r}$, and $P_{\mathrm{noise}}$ are expressed in dBm. The final throughput proxy is generated as

(21) $$T\left(d,t\right)=B\log_{2}\left(1+\gamma \right)\exp\;\left(-\kappa N_{75}\left(t\right)\right),$$

where *B* is the channel bandwidth in Hz, κ is the dimensionless congestion-decay coefficient, and $N_{75}\left(t\right)$ is the number of vehicles within a 75 m radius at time step *t*. The resulting value is expressed in bit/s and is divided by $10^{9}$ when reported in Gbps.

In the simulation, $B=10^{10}$ Hz, corresponding to a channel bandwidth of 10 GHz. Since the effective spectral efficiency ($\log_{2}\left(1+\gamma \right)\exp\left(-\kappa N_{75}\left(t\right)\right)$) can exceed 1 bit/s/Hz, the generated throughput may exceed 10 Gbps.

The primary objective of this study is not to reproduce packet-level TCP behavior itself but to evaluate the proposed budget-constrained vehicle selection, map maintenance, and uncertainty-aware estimation framework under spatially and temporally varying communication conditions. The adopted Shannon-capacity-based throughput proxy provides a controlled and reproducible communication-performance field whose spatial variation is determined by location-dependent propagation conditions and whose temporal variation is influenced by local vehicle density. Together with the budget-constrained sensing process, this setting provides the spatial and temporal variability and sparse-observation conditions required to evaluate the core methodological mechanisms investigated in this work.

**Figure 2:**
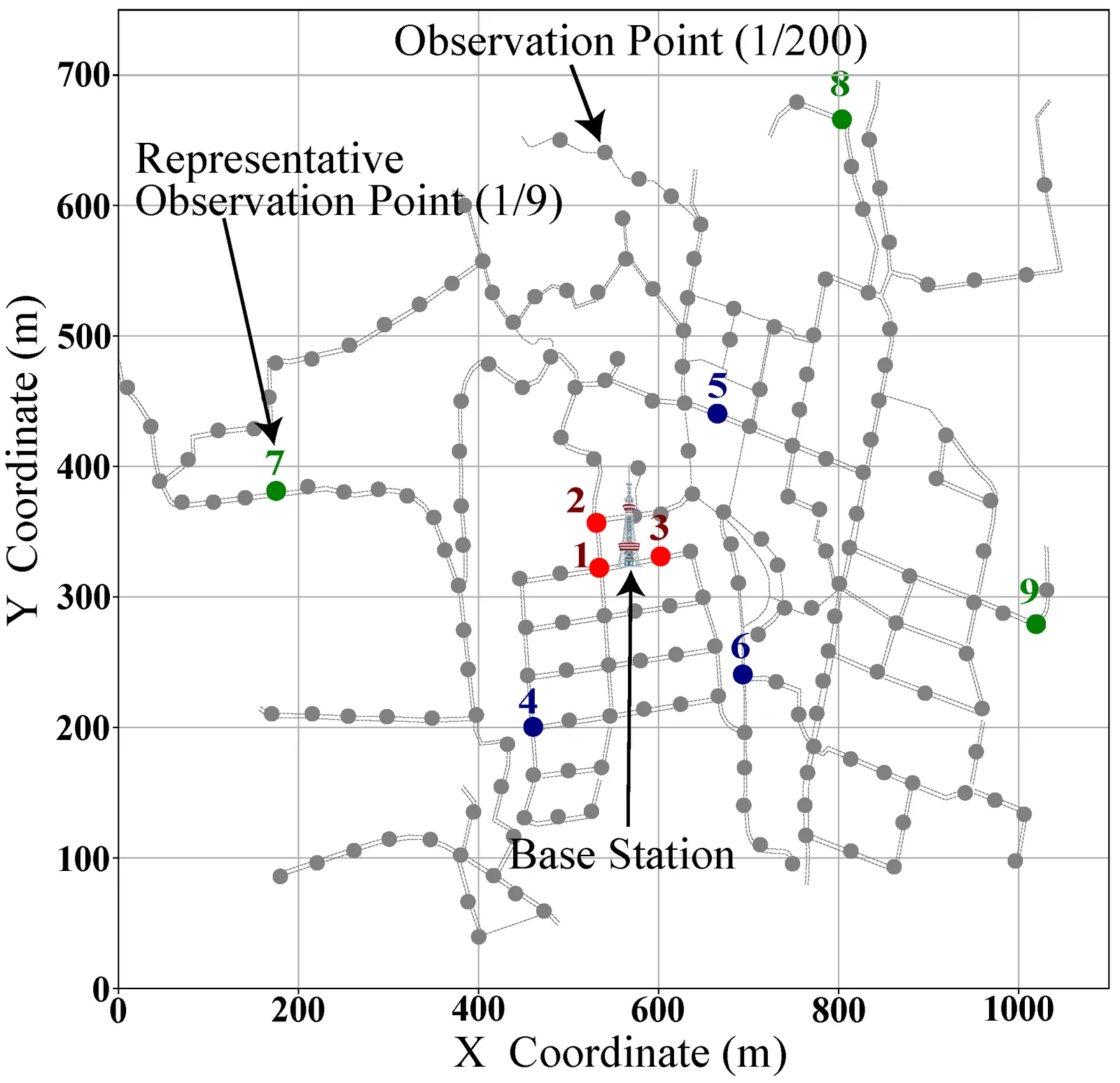
Spatial distribution of observation points and the base station.

As shown in Figure 2, 200 fixed observation points are distributed across the road network, and a base station is located at the center of the area. The proposed DISMAP algorithm is implemented in Python 3.9.

The online vehicle selection module exhaustively evaluates candidate vehicle subsets under a sensing budget of K=2 and selects the subset that minimizes global discoverability.

Let $M_{t}$ denote the number of available vehicles at time step *t*. The proposed method evaluates ∑k=1KMtk nonempty candidate subsets. With K=2 and $M_{t}\leq 32$, at most, 321+322=528 candidate subsets are examined at each time step. Exhaustive selection is preferred in the considered operating range because it directly identifies the subset that minimizes the proposed history-dependent global discoverability objective, whereas a greedy information-gain method would provide an approximate solution without an established submodularity guarantee for this objective.

Standard GPR was retained because the rolling window contains measurements from only the most recent H=50 time steps. Since, at most, K=2 vehicles are selected at each time step, the training set contains, at most, KH=100 samples. This bounded training-set size makes standard GPR computationally manageable while preserving the exact posterior mean and variance used in the uncertainty-calibration procedure. The current implementation is therefore intended for K≤2, $M_{t}\leq 32$, and H=50. For larger vehicle populations, sensing budgets, or training windows, greedy selection and sparse or online GP methods can be adopted.

In our implementation on a local machine equipped with an Intel Core i9-14900KF CPU (3.20 GHz) and 128 GB RAM running Windows 11 (GPU not used), the vehicle selection step takes, on average, approximately 2.4 ms per time step.

For uncertainty calibration, the ground-truth throughput values of all 200 fixed observation points during the first 25 time steps are used for PICP-based calibration of (a,b,c). The calibrated parameters are then fixed, and the remaining 175 time steps are used for prediction and final evaluation over the same 200 observation points. Thus, the calibration and evaluation periods are temporally non-overlapping, and no ground-truth throughput values from the evaluation period are used for calibration. The complete simulation settings are summarized in Table 1.

### 5.2. Convergence Analysis of DISMAP

**Figure 3:**
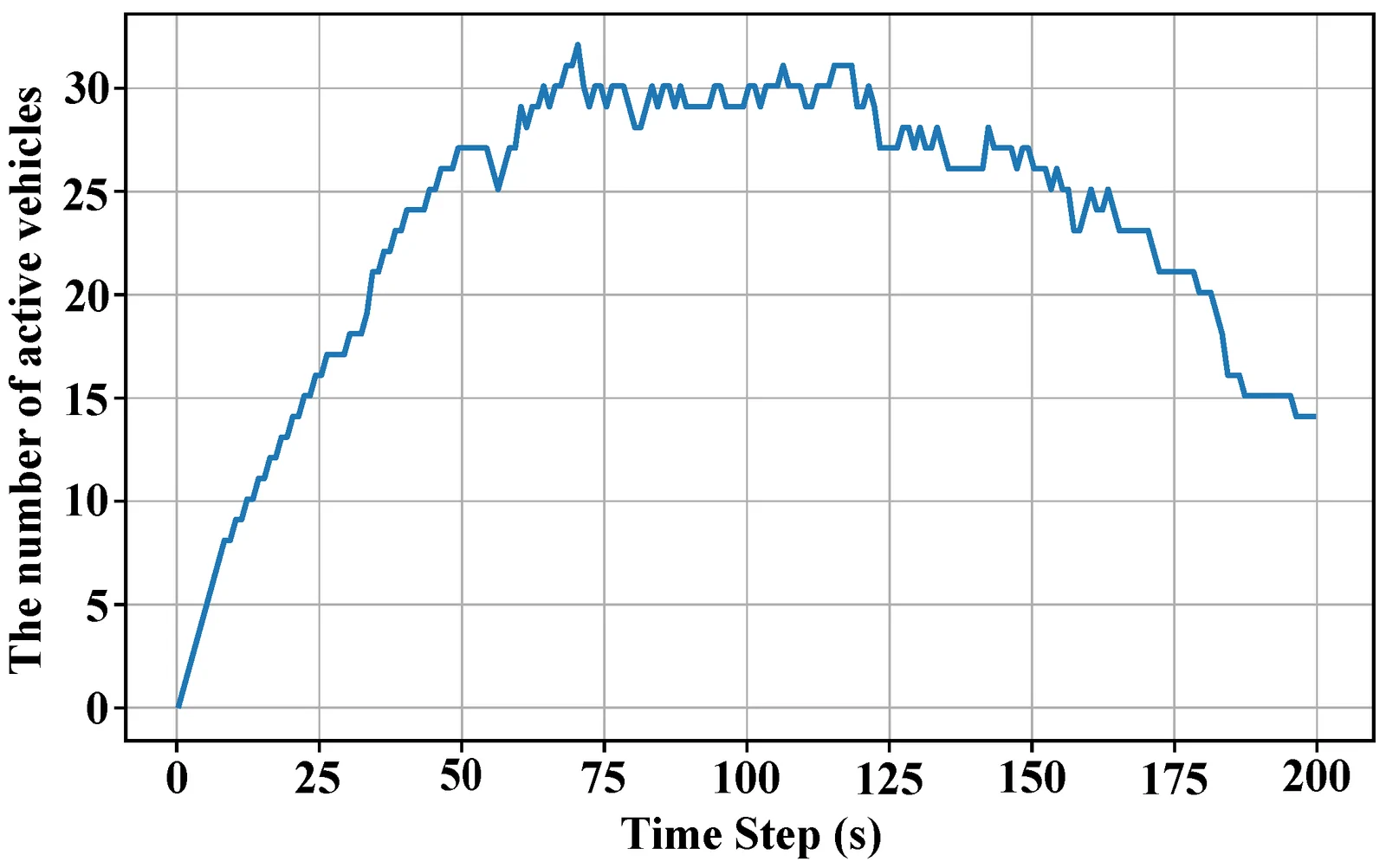
Available vehicles per time step.

Figure 3 shows the number of vehicles available at each time step. The vehicle count varies over time, increasing in the early stage, remaining relatively stable in the middle stage, and gradually decreasing toward the end. This time-varying availability is imposed to emulate a general IoV crowdsensing setting.

**Figure 4:**
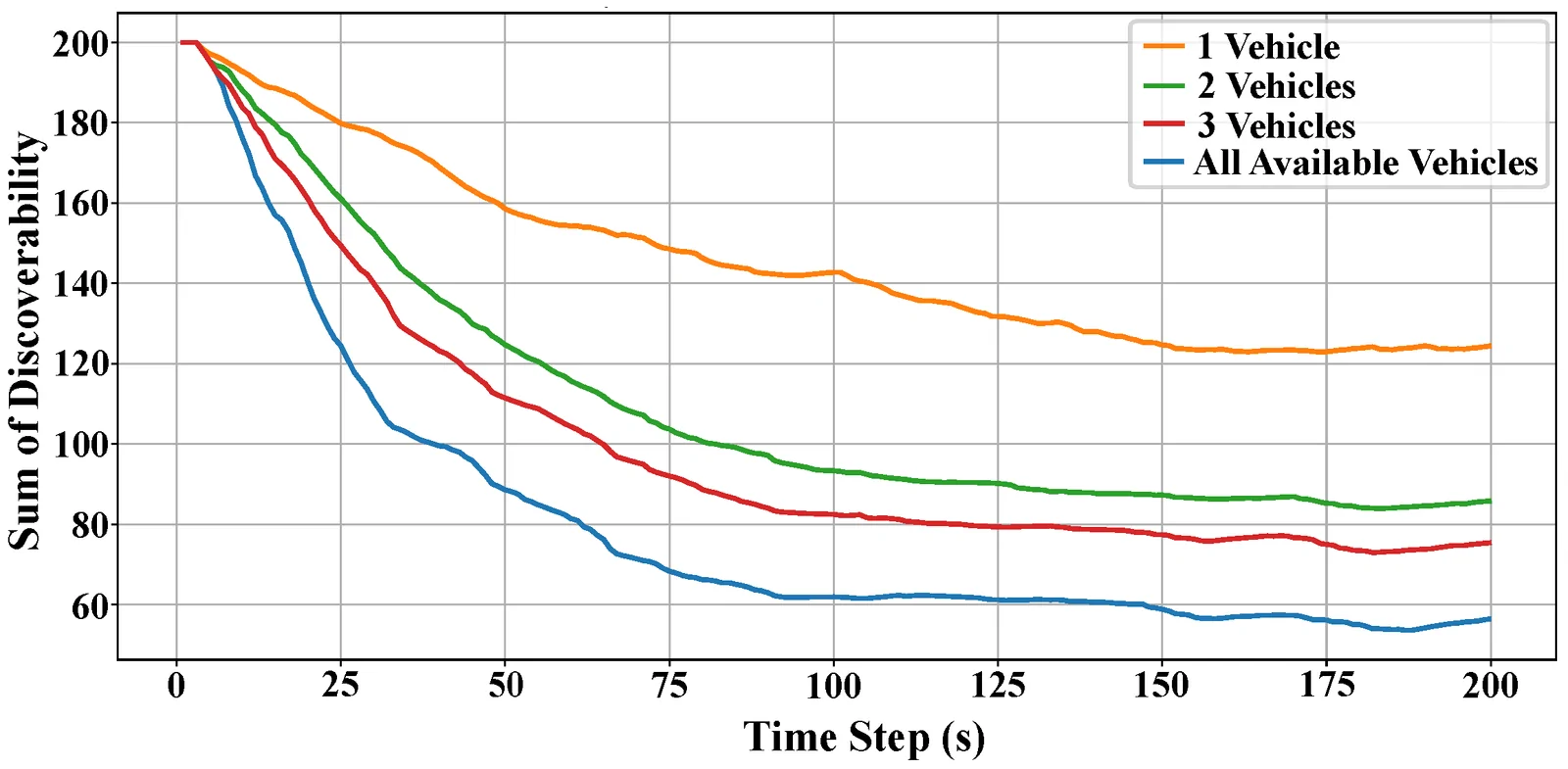
Discoverability-guided sensing performance without ground truth under different vehicle quantities.

Figure 4 illustrates the temporal evolution of TCP throughput map discoverability under different vehicle selection quantities. As the number of selected vehicles increases, the overall discoverability decreases more rapidly, indicating that the sensing process becomes more effective. In particular, the reduction from one to two vehicles is substantial, whereas the additional improvement from two to three vehicles is less pronounced, suggesting diminishing returns beyond two vehicles. Motivated by this cost–performance trade-off, we set the maximum number of selected vehicles at each time step to $K^{t}=2$ in the following DISMAP convergence analysis.

**Figure 5:**
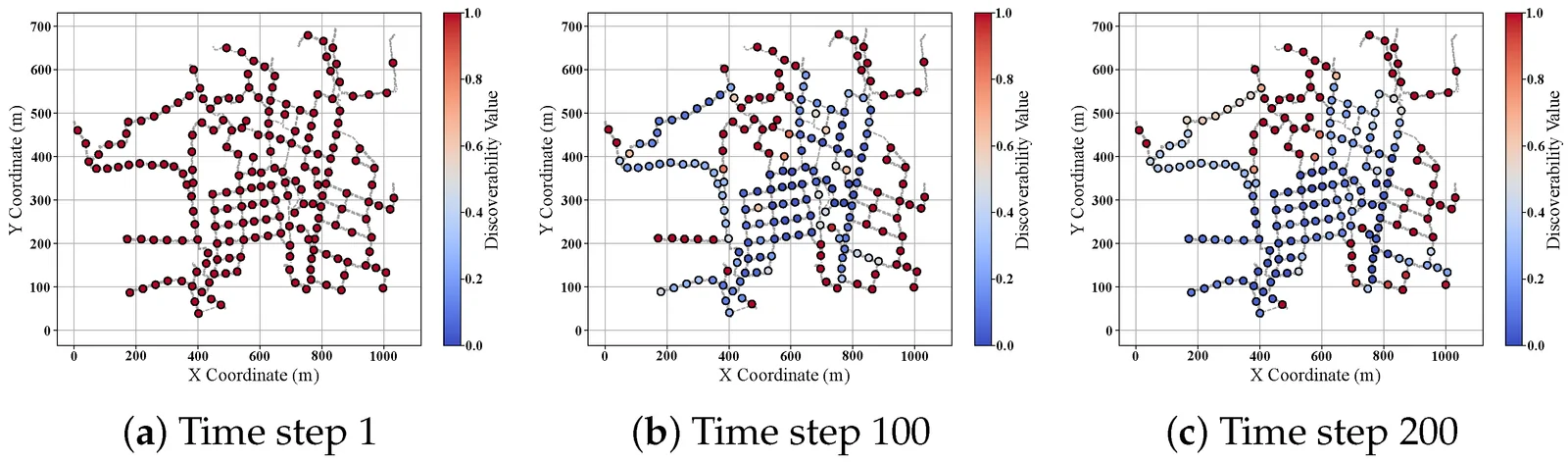
TCP throughput discoverability across the road network at t = 1, 100, and 200.

Figure 5 illustrates the discoverability maps at time steps 1, 100, and 200, where discoverability is the proposed decision signal used to guide the selection of the sensing-vehicle combination in DISMAP. It quantifies the potential information gain of each observation point and is incorporated into the selection objective to choose vehicle sets that most effectively improve throughput-map prediction. From time steps 1 to 200, the spatial pattern of discoverability evolves as measurements are collected and the model is updated: discoverability decreases in regions where predictive uncertainty has been sufficiently reduced, implying diminishing marginal information gain, while it remains high in areas that still lack informative observations, indicating persistent information gaps. Overall, at time step 200, the total discoverability across all observation points decreases to 85.82.

**Figure 6:**
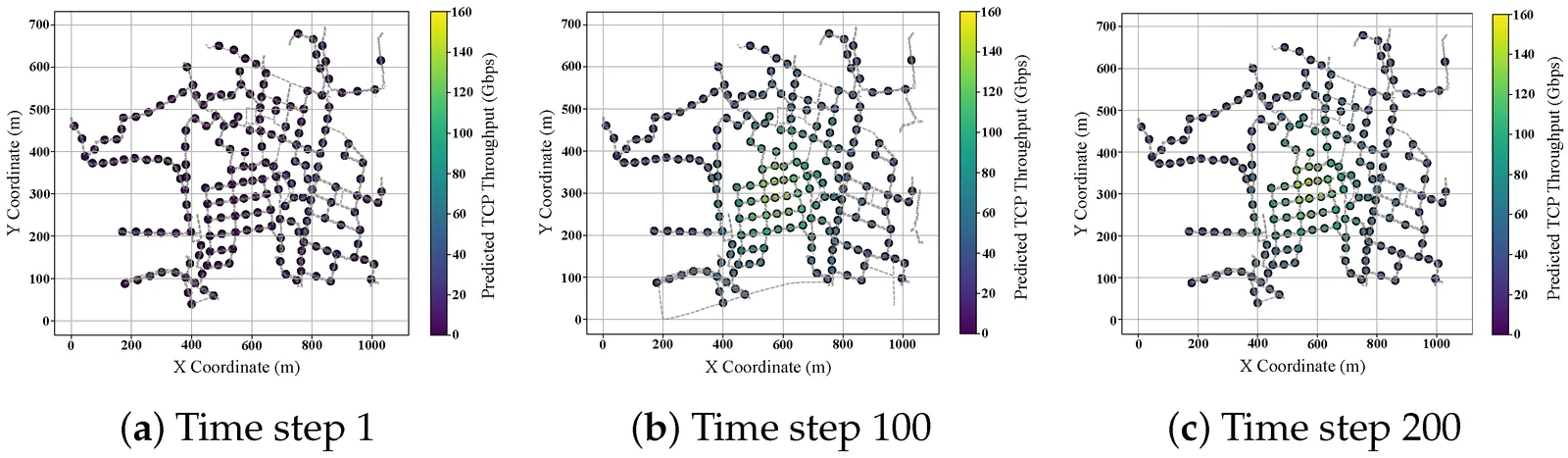
Predicted TCP throughput across the road network at t = 1, 100, and 200.

Figure 6 illustrates the predicted mean TCP throughput maps generated by DISMAP at time steps 1, 100, and 200, with the sensing vehicles selected as a combination guided by spatio-temporal discoverability. At time step 1, the prediction is still dominated by local structures due to the limited number of available measurements, and the global spatial pattern is not yet fully formed. By time step 100, as observations accumulate and information gaps are progressively filled under discoverability-guided selection, the spatial structure of the throughput field becomes noticeably more complete and clearer. By time step 200, the predicted throughput distribution further stabilizes and exhibits a consistent spatial gradient: relatively higher throughput values appear mainly in the core region of road networks and gradually decrease toward the periphery. This demonstrates that the proposed discoverability mechanism continuously highlights under-informed regions over time and provides an explicit optimization direction for vehicle-set selection. By allocating sensing opportunities toward locations with higher potential information gain, DISMAP progressively reconstructs the TCP-throughput landscape as observations accumulate.

**Figure 7:**
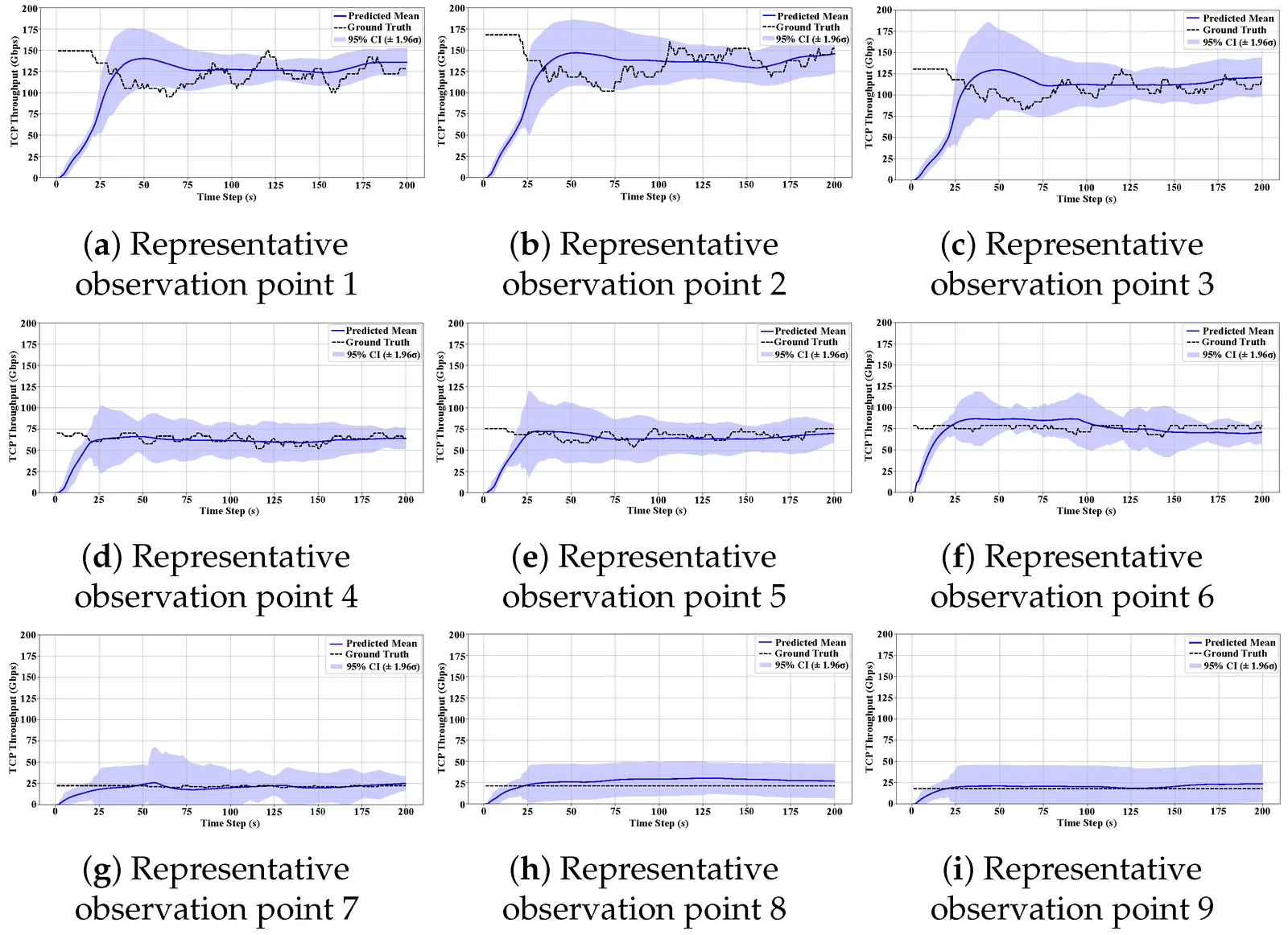
Prediction results at nine representative observation points (1–9), showing the predicted mean, 95% confidence intervals, and ground truth over time.

Figure 7 presents the predicted mean TCP throughput, ground truth, and 95% confidence intervals (CIs) [38] over time for nine representative observation points (Points 1–9), grouped into three categories based on their spatial distance from the base station, with their locations shown in Figure 2.

The observation points presented in Figure 7a–c (Points 1–3) are located close to the base station, where signal quality is strongest and throughput is generally high. However, due to the high density of vehicles and frequent changes in topology, these points also exhibit significant throughput fluctuations. Our framework accurately captures such variations by predicting wider confidence intervals, reflecting increased uncertainty.

The observation points presented in Figure 7d–f (Points 4–6) are in intermediate regions, where the vehicle density and channel conditions are relatively stable. The predicted throughput closely aligns with the ground truth, and the confidence intervals remain consistently narrow, indicating robust estimation under typical traffic conditions.

The observation points presented in Figure 7g–i (Points 7–9) are located in distant regions far from the base station, where vehicles are infrequent and throughput values remain consistently low. The predicted mean throughput remains stable and accurate, with tight confidence intervals. In particular, although Points 8 and 9 are never directly observed, DISMAP still generates plausible predictions with reasonable uncertainty levels, demonstrating its strong generalizability.

These results collectively demonstrate the advantages of the DISMAP framework: it enables accurate prediction of the TCP throughput mean at each observation point while adaptively modeling uncertainty in response to spatial throughput dynamics. This approach effectively reduces prediction uncertainty without compromising confidence coverage.

### 5.3. Comparative Performance Analysis of DISMAP

To comprehensively evaluate the proposed DT-based TCP throughput map construction framework, we conduct experiments from four perspectives:

1. Effect of sensing budget (vehicle quantity):
  We vary the number of selected vehicles per time step (one, two, three, and all available vehicles) and evaluate the resulting prediction performance, revealing the trade-off between sensing cost and the accuracy of map construction.
2. Effect of traffic density: We evaluate DISMAP under sparse, moderate, and congested traffic scenarios to examine its robustness and generalization capability under different traffic densities.
3. Comparison of vehicle selection objectives: We compare DISMAP with three objective-driven baselines to evaluate the effectiveness of the proposed vehicle selection objective.
4. Comparison of mapping methods: We first conduct an ablation study on the uncertainty calibration module, then compare DISMAP with MLP-based mapping methods to evaluate the effectiveness of the proposed mapping strategy under practical data constraints.

We use the following three metrics to evaluate model performance. To ensure consistent and meaningful comparison, all evaluation metrics are reported over a stable interval from time step 26 to time step 200, excluding early-stage predictions where limited initial observations may lead to unstable estimates.

Mean Absolute Error (MAE)

MAE quantifies the average absolute difference between the predicted and ground-truth throughput values across all observation points and time steps:

(22) $$\mathrm{MAE}=\frac{1}{N(T-25)}\sum_{t=26}^{T}\sum_{n=1}^{N}\left|\hat{\mu }\left(g_{n}^{t}\right)-T\left(g_{n}^{t}\right)\right|$$

where $\hat{\mu }\left(g_{n}^{t}\right)$ denotes the predicted mean throughput and $T(g_{n}^{t})$ denotes the ground-truth throughput.

Mean Standard Deviation (MSTD)

MSTD characterizes the average predictive uncertainty by computing the mean of the predicted standard deviations across the entire spatio-temporal domain:

(23) $$\mathrm{MSTD}=\frac{1}{N(T-25)}\sum_{t=26}^{T}\sum_{n=1}^{N}\sigma \left(g_{n}^{t}\right)$$

where $\sigma (g_{n}^{t})$ is the predicted standard deviation at observation point $g_{n}$ and time *t*.

Prediction-Interval Miss Rate (PIMR)

We first define the Mean Prediction-Interval Coverage Probability (MPICP), which assesses the overall calibration quality by calculating the proportion of ground-truth values that fall within the predicted 95% prediction intervals:

(24) $$\mathrm{MPICP}=\frac{1}{N(T-25)}\sum_{t=26}^{T}\sum_{n=1}^{N}I\left[T\left(g_{n}^{t}\right)\in \left[\hat{\mu }\left(g_{n}^{t}\right)\pm 1.96\cdot \sigma \left(g_{n}^{t}\right)\right]\right]$$

where I[·] is the indicator function that returns 1 if the condition holds and 0 otherwise.

To quantify the proportion of missed predictions, we define the PIMR as follows.

(25) PIMR=1−MPICP

Ideally, a well-calibrated model should yield a PIMR close to 0.05, meaning that approximately 95% of the ground-truth values fall within the prediction intervals. A PIMR above 0.05 indicates undercoverage and potentially overly narrow intervals, whereas a PIMR substantially below 0.05 may indicate overcoverage and overly conservative intervals. Therefore, interval calibration is assessed using PIMR, while interval sharpness is assessed using MSTD, and the two metrics are interpreted jointly.

### 5.3.1. Effect of Sensing Budget (Vehicle Quantity)

**Figure 8:**
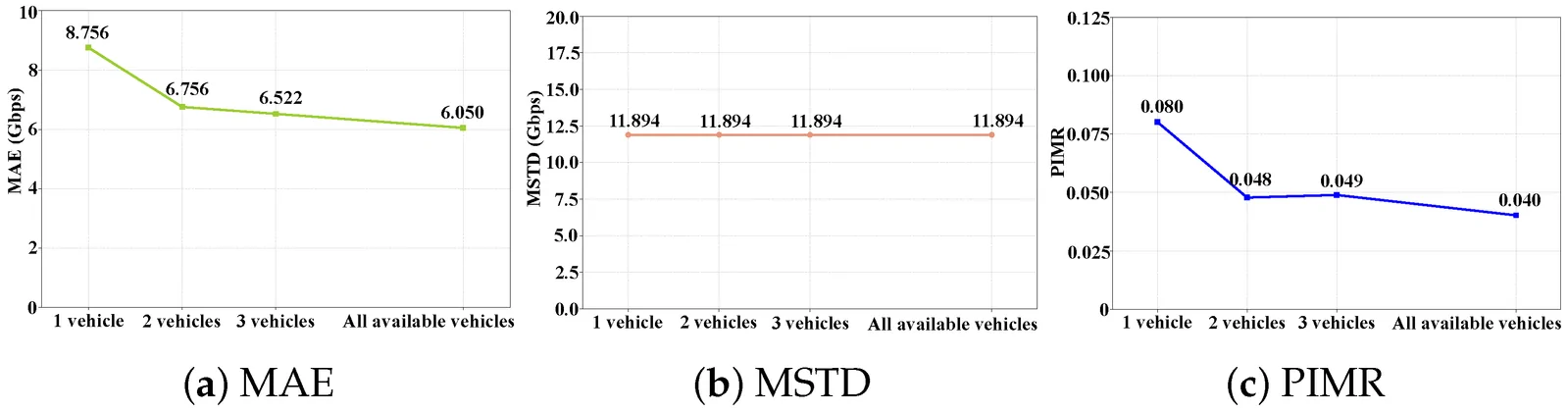
Performance comparison across different vehicle selection quantities over time steps 26–200.

Figure 8 presents a performance comparison under different numbers of selected vehicles, evaluated in terms of MAE, MSTD, and PIMR over time steps 26 to 200. As shown in Figure 8a, increasing the number of selected vehicles significantly reduces the MAE, with the most notable improvement observed when increasing from one to two vehicles. Notably, this cost–performance pattern is well captured by our discoverability metric in Figure 4: as more vehicles are selected, the total discoverability decreases faster, and the largest reduction likewise occurs when moving from one to two vehicles, indicating that discoverability can serve as a reliable proxy for sensing effectiveness without requiring ground truth. The MSTD shown in Figure 8b remains constant across all vehicle quantities due to the use of a fixed density-aware uncertainty calibration mechanism based on vehicle density. This indicates that our uncertainty modeling remains robust under different sensing configurations. In Figure 8c, the PIMR changes from 0.080 with one selected vehicle to 0.040 when all available vehicles are used, approaching the nominal value of 0.05. However, the marginal change becomes limited beyond the selection of two vehicles. Considering prediction accuracy, interval sharpness, calibration, sensing overhead, and computational cost jointly, we select two vehicles per time step in the subsequent experiments.

### 5.3.2. Impact of Traffic Density

**Figure 9:**
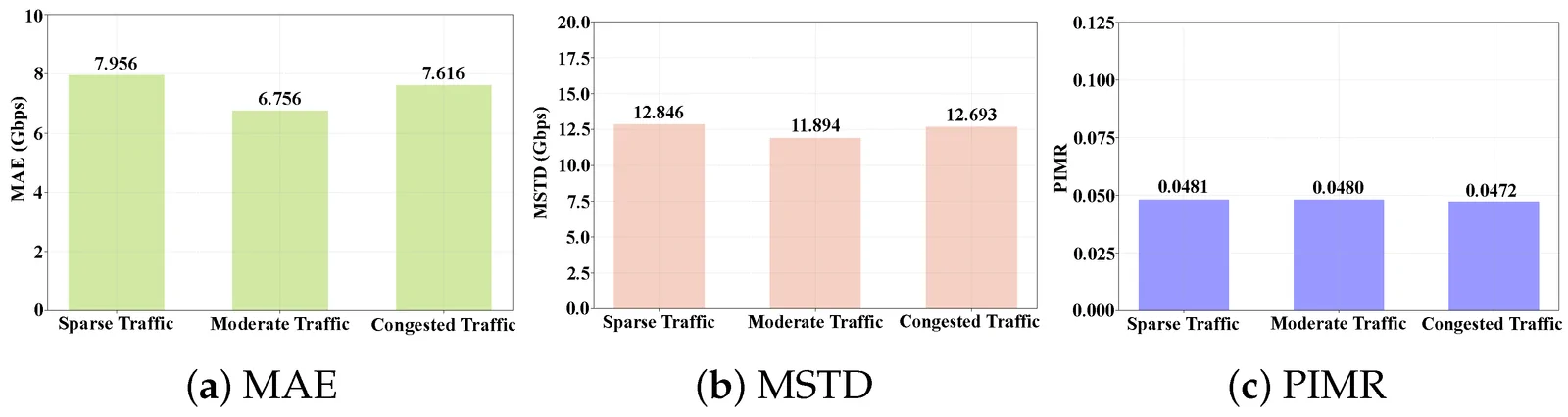
Performance comparison of DISMAP under three traffic-density scenarios over time steps 26–200.

Figure 9 evaluates the performance of DISMAP under three representative traffic density scenarios—namely, sparse, moderate, and congested traffic—over time steps 26 to 200. The average numbers of available vehicles over time steps 26–200 are 6.34 and 24.75 for the sparse and congested traffic scenarios, respectively. The corresponding maximum numbers of available vehicles are 8 and 32, respectively, indicating clearly different candidate-vehicle scales across the three scenarios. As shown in Figure 9a, DISMAP achieves the lowest MAE in the moderate traffic scenario (6.756 Gbps), while the MAE slightly increases in the sparse traffic scenario (7.956 Gbps) and the congested traffic scenario (7.616 Gbps). This is because in sparse traffic, fewer vehicles are available for selection, which limits the amount and spatial diversity of sensing data. In contrast, in congested traffic, the TCP throughput fluctuates more strongly due to more severe channel contention, cell-load variation, and vehicle-density changes. A similar trend can be observed in Figure 9b. DISMAP achieves the lowest MSTD in the moderate traffic scenario (11.894 Gbps), while higher MSTD values are observed in sparse traffic (12.846 Gbps) and congested traffic (12.693 Gbps). This indicates that uncertainty increases when the available sensing information is insufficient or when the communication environment is highly dynamic. Nevertheless, Figure 9c shows that the PIMR values remain close to the nominal value of 0.05 across all three scenarios, with values of 0.0481, 0.0480, and 0.0472 under sparse, moderate, and congested traffic, respectively.

Overall, these results show that DISMAP maintains stable performance across varying traffic densities, indicating its robustness and effectiveness, even as the number of candidate vehicles increases in denser IoV scenarios.

### 5.3.3. Comparison of Vehicle Selection Objectives

**Figure 10:**
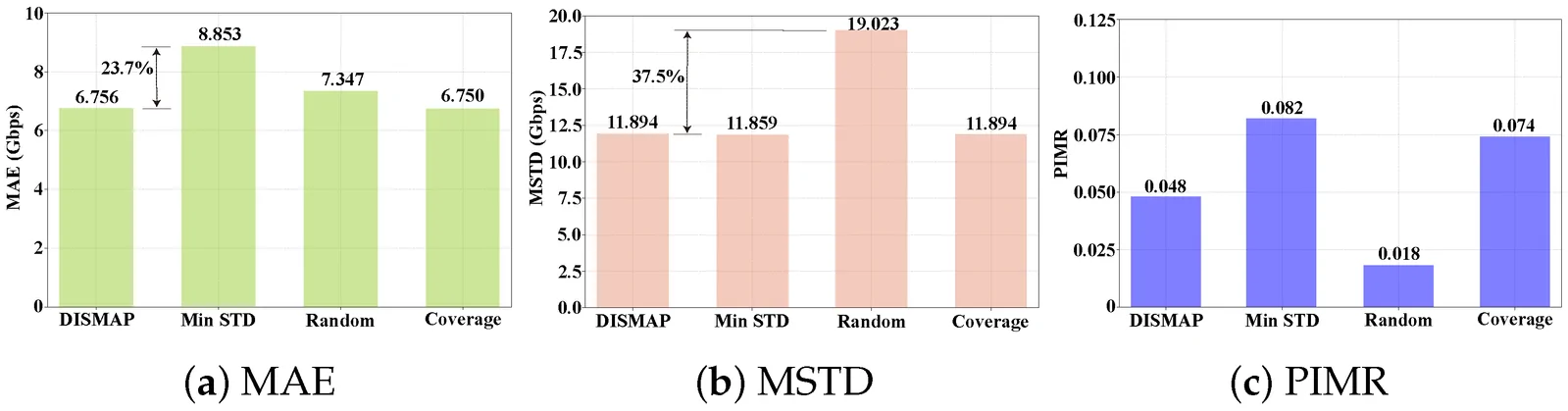
Performance comparison among different vehicle selection objective functions over time steps 26–200.

Figure 10 presents a performance comparison among different objective functions of vehicle selection, evaluated in terms of MAE, MSTD and PIMR over time steps 26 to 200. The compared objective functions include the following.

- Min STD: At each time step, this baseline chooses the vehicle set that minimizes $\sum_{n\in G}\sigma_{n}^{t}$, where $\sigma_{n}^{t}$ denotes the predicted standard deviation of the GPR predictive distribution at observation point $g_{n}$. It focuses solely on uncertainty reduction without explicitly enforcing spatial coverage [39].
- Coverage: A coverage-driven baseline that selects a vehicle set to maximize spatial coverage (via a 100 m sensing radius) in order to reduce spatial sparsity and potentially improve prediction accuracy, without explicitly modeling or minimizing predictive uncertainty [40].
- Random: A baseline approach where a vehicle set is randomly selected at each time step, without any optimization objective. It serves as a reference for uninformed selection.

As shown in Figure 10a, DISMAP and Coverage achieve the lowest MAE (6.756 Gbps and 6.750 Gbps, respectively), while Min STD results in the highest MAE (8.853 Gbps). In particular, DISMAP achieves a 23.7% reduction in MAE compared to Min STD, demonstrating that minimizing predictive uncertainty alone does not ensure accurate throughput estimation and that joint modeling of precision and coverage is more effective. Random selection performs moderately (7.347 Gbps), confirming the necessity of informed strategies. As shown in Figure 10b, both DISMAP and Coverage produce average MSTD values of 11.894 Gbps, which is comparable to Min STD (11.859 Gbps) but significantly lower than Random (19.023 Gbps). This represents a 37.5% reduction in predictive uncertainty compared to Random, highlighting the robustness of informed selection strategies under heterogeneous noise. Figure 10c shows that DISMAP achieves a PIMR of 0.048, which is close to the nominal value of 0.05. Although Random produces a numerically lower PIMR of 0.018, its deviation from the nominal value is substantially larger than that of DISMAP (0.032 versus 0.002). Together with its considerably higher MSTD of 19.023 Gbps, this result indicates that Random produces overly wide and conservative prediction intervals. Coverage yields a PIMR of 0.074, indicating undercoverage. In summary, although Coverage and Min STD achieve marginally lower MAE and MSTD values, respectively, DISMAP provides a favorable overall balance among prediction accuracy, interval sharpness, calibration, and spatial representativeness.

### 5.3.4. Comparison of Mapping Methods

**Figure 11:**
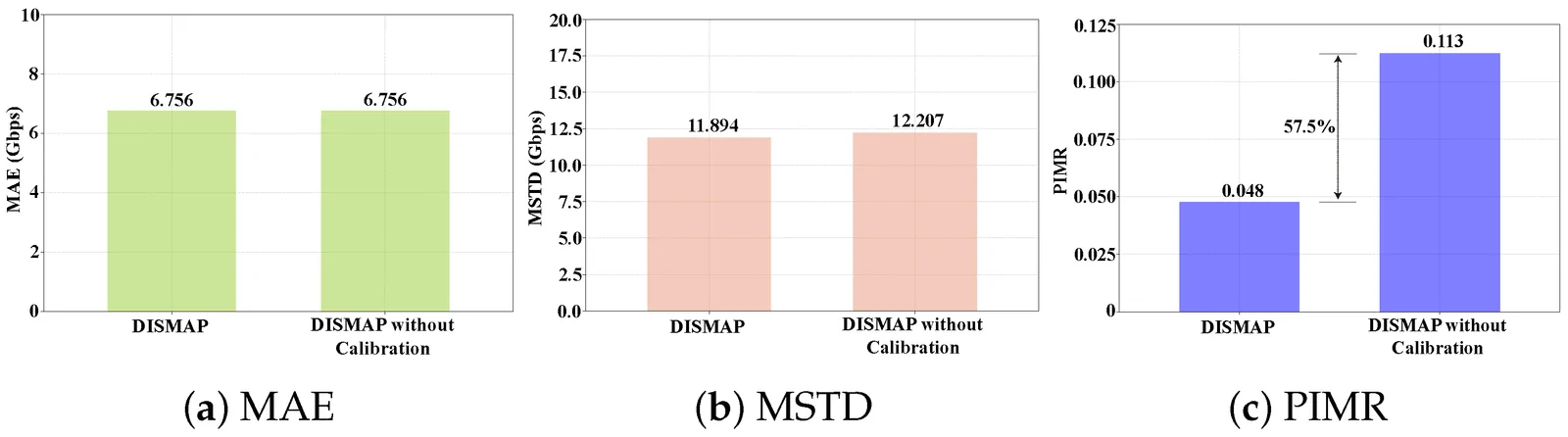
Performance comparison between DISMAP and DISMAP without calibration over time steps 26–200.

Figure 11 presents an ablation study on the uncertainty calibration module in DISMAP, evaluated in terms of MAE, MSTD, and PIMR over time steps 26 to 200. To isolate the effect of this module, both methods use the same spatio-temporal discoverability-based vehicle selection module, while DISMAP without calibration removes the uncertainty calibration mechanism from the GPR mapping module. As shown in Figure 11a, DISMAP and DISMAP without calibration achieve the same MAE of 6.756 Gbps, indicating that the calibration mechanism does not affect the predicted mean throughput. However, DISMAP provides better-calibrated uncertainty estimates. Specifically, Figure 11b shows that DISMAP achieves a slightly lower MSTD than DISMAP without calibration (11.894 vs. 12.207 Gbps). Figure 11c shows PIMR values of 0.048 and 0.113, respectively. The PIMR of DISMAP is substantially closer to the nominal value of 0.05, indicating that the calibration mechanism improves interval calibration while preserving the predictive accuracy of the base GPR model. By adjusting the predictive standard deviation according to local traffic-density information, DISMAP better aligns the prediction intervals with the target coverage level.

**Figure 12:**
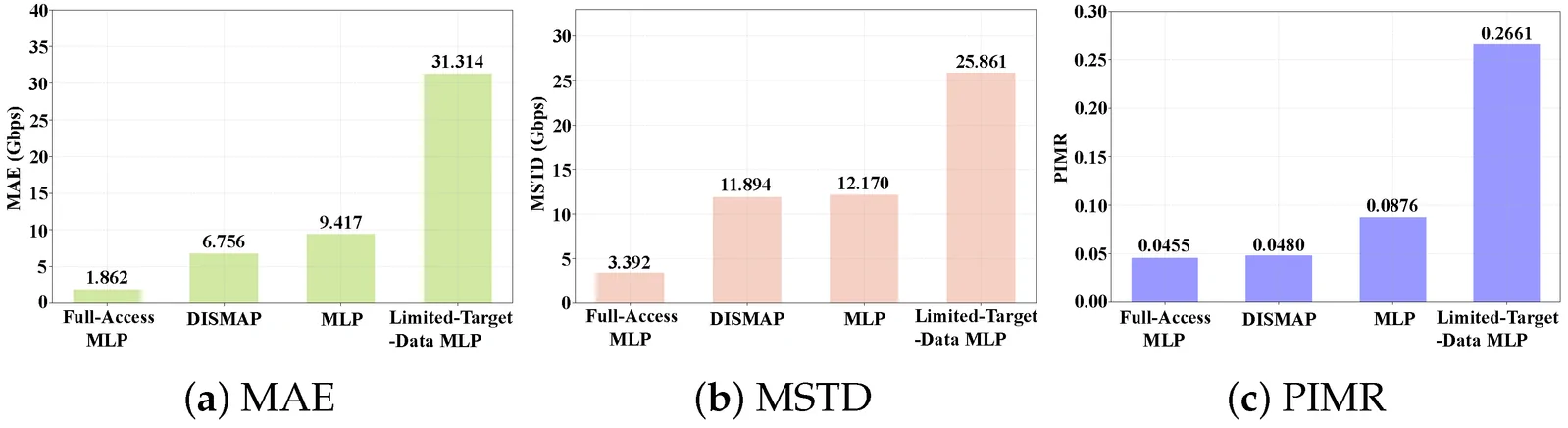
Performance comparison of DISMAP and MLP-based mapping methods over time steps 26–200.

We further compare DISMAP with probabilistic Multi-Layer Perceptron (MLP)-based mapping methods under different data-availability and adaptation settings. Figure 12 presents the performance comparison over the 175 evaluation time steps following the initial 25-time-step calibration period. The compared methods are defined as follows.

- Full-Access MLP: An optimistic oracle reference that assumes access to the complete ground-truth throughput maps of all observation points in the target scenario. At each time step, the model is updated using the complete target-scenario maps available from the preceding time steps and is subsequently used to predict the next throughput map. This setting provides a favorable reference for MLP performance but is not practical in real IoV systems because complete target-scenario throughput maps are generally unavailable.
- External-Data MLP: A probabilistic MLP trained offline using three external traffic datasets with traffic-density conditions, which are independent of the target evaluation dataset. Each external dataset contains 200 time steps over the same 200 observation points, providing up to 40,000 spatio-temporal samples per dataset. After offline training, the model parameters remain fixed while predicting the target traffic scenario, and no target-scenario throughput ground truth is used for model training [41].
- Limited-Target-Data MLP: A small-sample baseline trained using only the target-scenario samples available during the initial 25-time-step calibration period. The same 200 fixed observation points and the initial 25-time-step data budget used in the calibration stage of DISMAP are adopted After this initial training stage, the model parameters remain fixed, and the trained MLP is used to predict the remaining 175 time steps of the target scenario.

All three MLP variants employ the same probabilistic neural-network architecture. The input vector consists of the observation-point coordinates, local vehicle density, current uncertainty value, normalized time index, and spatially weighted throughput derived from the selected vehicles. The network contains two fully connected hidden layers with 128 and 64 neurons, respectively, both using ReLU activation. Two separate output heads estimate the predictive mean and log variance of throughput. The input features are standardized before training. The models are trained using the Gaussian negative log-likelihood loss and the Adam optimizer with a learning rate of $10^{-3}$ for 200 full-batch epochs. The Full-Access MLP is updated at every target-scenario time step, whereas the External-Data MLP and Limited-Target-Data MLP are trained only once and remain fixed during their corresponding evaluation periods.

As shown in Figure 12, Full-Access MLP serves only as an infeasible upper-bound reference for MLP performance, since it performs rolling training and prediction using the complete ground-truth throughput information of all observation points. Compared with the standard MLP, DISMAP reduces the MAE from 9.417 Gbps to 6.756 Gbps and the MSTD from 12.170 Gbps to 11.894 Gbps. Its PIMR of 0.0480 is also substantially closer to the nominal value of 0.05 than the MLP result of 0.0876, indicating better interval calibration. Furthermore, compared with Limited-Target-Data MLP, DISMAP provides a more favorable balance between mapping accuracy, interval sharpness, and calibration under the considered small-sample setting.

These results indicate that DISMAP does not rely on the complete ground-truth information assumed by Full-Access MLP. Under the considered data-availability and adaptation settings, DISMAP provides a favorable balance among prediction accuracy, interval sharpness, and calibration.

## 5.4. Limitations

While the adopted Shannon-capacity-based throughput proxy does not reproduce all practical TCP dynamics, it provides the spatial and temporal variations required to evaluate the core research question of this study—namely, whether the proposed vehicle-selection, map-maintenance, and uncertainty-aware estimation framework can reconstruct and maintain a spatial communication-performance map under limited sensing resources. Nevertheless, the current model does not explicitly capture packet-level TCP effects such as packet loss, retransmissions, protocol overhead, measurement errors, message losses, or time-varying mobile-network parameters. Therefore, the present experiments should be interpreted as a controlled validation of the proposed map-maintenance methodology rather than a direct characterization of actual end-to-end TCP performance. Packet-level TCP simulation and real vehicular measurements will be considered in future work.

## 6. Conclusions

This paper addressed online TCP throughput map maintenance under sparse vehicular observations and limited sensing budgets in dynamic Internet of Vehicles (IoV) environments. We presented Discoverability-aware and Statistical Mapping (DISMAP), a sensing-and-mapping method for a digital twin (DT)-assisted vehicular sensing architecture. DISMAP combines spatio-temporal discoverability-based vehicle selection with Gaussian Process Regression (GPR)-based probabilistic map updating. The discoverability component improves spatial representativeness under limited sensing budgets, while the statistical mapping component estimates TCP throughput and adjusts the predictive standard deviation using local vehicle density. Simulation results indicate that DISMAP provides a favorable balance among prediction accuracy, interval sharpness, calibration, and spatial representativeness under a limited sensing budget. The proposed method also maintains stable performance across different traffic-density conditions.

## Figures and Tables

**Table 1:** Simulation settings.

Parameter	Value
Number of Observation Points (*N*)	200
Discoverability of Observation Point *D*	0.0–1.0
Initial Discoverability ($D(g_{n}^{1})$)	1.0
Available Vehicles per Time Step (*t*)	1–32
Maximum Selected Vehicles per Step (*K*)	1, 2, 3, All Available Vehicles
Time Steps (*T*)	1–200
Base-Station Configuration	1, (575 m, 320 m)
Local Vehicle-Counting Radius	75 m
Logging Frequency	1 Hz
Channel Bandwidth (*B*)	10 GHz
Transmit Power ($P_{\mathrm{tx}}$)/Noise Floor ($P_{\mathrm{noise}}$)	30 dBm/−100 dBm
Path-Loss Exponent (η)/Additional Losses ($L_{\mathrm{abs}}+L_{\mathrm{sca}}$)	4.0/(15 dB + 5 dB)
Congestion-Decay Coefficient (κ)	0.05
GPR Window Size (*H*)	50
GPR Kernel Parameters	C=0.02,λ=100.0
Signal Variance ($\sigma_{f}^{2}$)	0.02
Spatial Length Scale (λ)	100
Spatial Length Scale ($l_{s}$)	60.0
Temporal Length Scale ($l_{t}$)	5.0
Scaling Parameters	a=2.0,b=1.2,c=0.35
Scaling Coefficient (β)	1.0

## Data Availability

The data supporting the findings of this study are contained within the article. Further inquiries can be directed to the corresponding author.
